# Realizing high-performance four active plasmonic filters using a single structure

**DOI:** 10.1038/s41598-024-80724-4

**Published:** 2024-11-27

**Authors:** Samar Elbialy, B. M. El-den, Eman Ashraf

**Affiliations:** Electronics and Communications Department, Faculty of Engineering, Delta University for Science and Technology, Gamasa, Egypt

**Keywords:** Plasmonic filter, Photonic integrated circuits, Full-width half maximum (FWHM), Hybrid plasmonic waveguides, FDTD algorithm, Engineering, Optics and photonics

## Abstract

This research aims to contribute significantly to the field of plasmonic filtering technology within modern optical communication systems. By focusing on the development of a high-performance, more compact, and efficient design, this study explores the potential of hybrid plasmonic filters to revolutionize optical filtering applications. The approach leverages an innovative active material with electrically tunable permittivity, allowing for dynamic control over the filter’s optical properties. The research specifically examines four types of filters: low-pass filters (LPF), high-pass filters (HPF), band-pass filters (BPF), and band-reject filters (BRF). These filters are designed to operate effectively across a broad wavelength range of 1200–1800 nm, achieving a transmittance exceeding 98% at the output port, while maintaining isolation with transmittance below 2% at the isolated ports. The structure demonstrates a FWHM of approximately 216 nm for the band-pass filter and approximately 223 nm for the band-reject filter, which are considered moderate values, ensuring the versatility and multifunctionality of the design. The ultra-compact size, with a footprint of just 21 µm^2^, makes these filters particularly advantageous for integration into space-constrained optical communication systems.

## Introduction

The burgeoning complexity and rapid advancement of modern optical communication systems, spurred by the increasing demand for data and technological progress, necessitate the continuous refinement of essential components to maintain efficient signal transmission and reception^1–7^. Within this landscape, plasmonic filters have emerged as critical elements in optical and telecommunication systems. These filters play a crucial role in various applications, including the selective isolation and transmission of specific wavelengths, enhancement of optical signal quality through noise and crosstalk reduction, and the integration of miniaturized photonic circuits where plasmonic filters work in tandem with other optical components. Additionally, plasmonic filters hold promise in biosensing applications, enabling real-time, label-free detection of biomolecules^8–10^.

Despite their significant potential, several obstacles hinder the widespread adoption of plasmonic filters. These challenges include substantial optical losses, which necessitate the development of strategies to reduce losses in plasmonic materials for improved filter efficiency; the complexity of fabrication, requiring advancements in techniques to achieve precise and cost-effective production; sensitivity to geometric variations, where fabrication imperfections can adversely affect filter performance; integration challenges. Particularly in combining plasmonic filters with conventional optical components; and the need for dynamic control and tunability, prompting the development of filters capable of real-time spectral response adjustments^11–14^.

Realizing High-performance Plasmonic Filters for optical communication system that can overcome the problem of low speed (small B.W), satisfies high transmission efficiency and appear in small foot print is considered the main goal of this work. The best way to match these advantages was using hybrid plasmonic waveguides which satisfies strong mode confinement and reduces the propagation loss and guarantees a cost-efficient mass production environment for such integrated photonic devices. Beside the using of electro-optic materials to realize the active mechanism. Ultimately, the success of electro-optical filtering depends on a few performance factors. Among which are a high frequency response, low power consumption, low optical losses, thermal stability, a small footprint, the FWHM, integrate ability with CMOS but also ease of fabrication and cost^15–17^.

Advancements in computational modeling and simulation techniques have played a crucial role in the optimization of plasmonic devices. Numerical simulations have been instrumental in refining plasmonic filter designs, particularly by highlighting the importance of geometry and material selection in achieving specific spectral properties. For this purpose, Lumerical FDTD Solutions, a powerful software package, is utilized to model and analyze the performance of the proposed active plasmonic filter. This software solves 3D Maxwell’s Equations using the Finite Difference Time Domain (FDTD) method, which is highly efficient in tackling complex problems and providing rapid analysis.

The FDTD algorithm is particularly well-suited for evaluating the proposed design due to its capability to efficiently process large datasets and deliver precise results in a short time frame. To ensure accurate simulation, the boundary conditions applied in these numerical methods are based on the differential form of Maxwell’s equations, with the implementation of a perfectly matched layer (PML). The PML acts as a layer of lossy material with a perfectly matched interface, effectively absorbing incident plane waves across all frequencies, angles, and polarizations, thereby minimizing reflection and enhancing the accuracy of the simulation^18^. The present work is structured as follows; Sect. (2) proposes realizing high-performance four active plasmonic filters and how they depend the (ON/OFF) cases of the used switching units then testing the performance of four’ type plasmonic filters through measuring some parameters^19–21^.

## Realizing high-performance active plasmonic filters

**Fig. 1:**
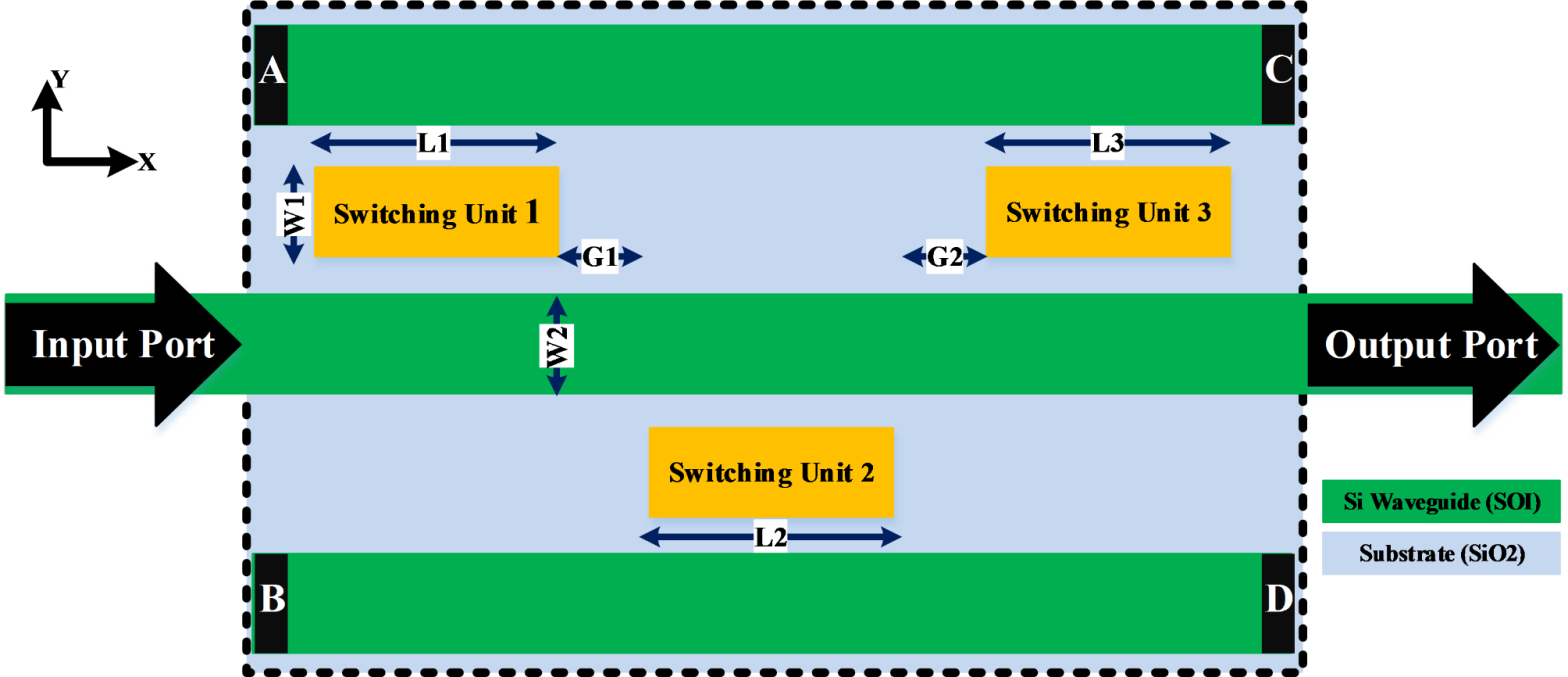
The 2D- XY view of the proposed plasmonic structure that realize the four filter’ types. The proposed structure consists of two identical Si arms with dimensions of 400 nm in width and 340 nm in height. The length of three switching units are L1, L2, and L3, and in the beginning of simulation they are adjusted to a value of μm. The three switching units are separated from the three waveguides by an equal distance of 150 nm in width and also the three switching units are separated from each other by a gap (G1 = G2) of 370 nm.

Plasmonic filters are essential in controlling the spectral characteristics of optical signals, functioning by manipulating surface plasmon polaritons (SPPs) to achieve selective wavelength transmission. This capability is critical for isolating specific wavelengths, which is essential for high-capacity data transmission in optical networks. The effectiveness of these filters is rooted in the distinctive property of plasmonic materials to support collective electron oscillations at metal-dielectric interfaces, which forms the foundation of their operation (Fig. 1).

The proposed plasmonic structure that realize the four filter’ types active plasmonic filter consists of two identical Si arms with dimensions of 400 nm in width and 340 nm in height, act as bus waveguides and their ports are isolated, and middle them a Si arm acts as input/output waveguide. Three switching units are sited between them have the same dimensions and materials. The length of three switching units are L1, L2, and L3 and in the beginning of simulation they are adjusted to a value of 6 μm according to approximation of the switching unit length can be set to an integer $\left(n\right)$ of the desired mode wavelength $\left(\lambda\right)$. The lengths of switching units are modified during the optimization process to $\left(\text{L}1=\text{L}3=3{\upmu}\text{m}\right)$ and $\left(\text{L}2=6{\upmu}\text{m}\right)$ in order to ensure a satisfactory power transfer between the input/output ports. The three switching units are separated from the three waveguides by an equal distance of 150 nm in width and also the three switching units are separated from each other by a gap (G1=G2) of 370 nm. All of the above are printed on a substrate from silicon dioxide. The filtering mechanism depends mainly on the switching units that control transferring the optical power from the input to the output ports.

### The performance of switching units

**Fig. 2:**
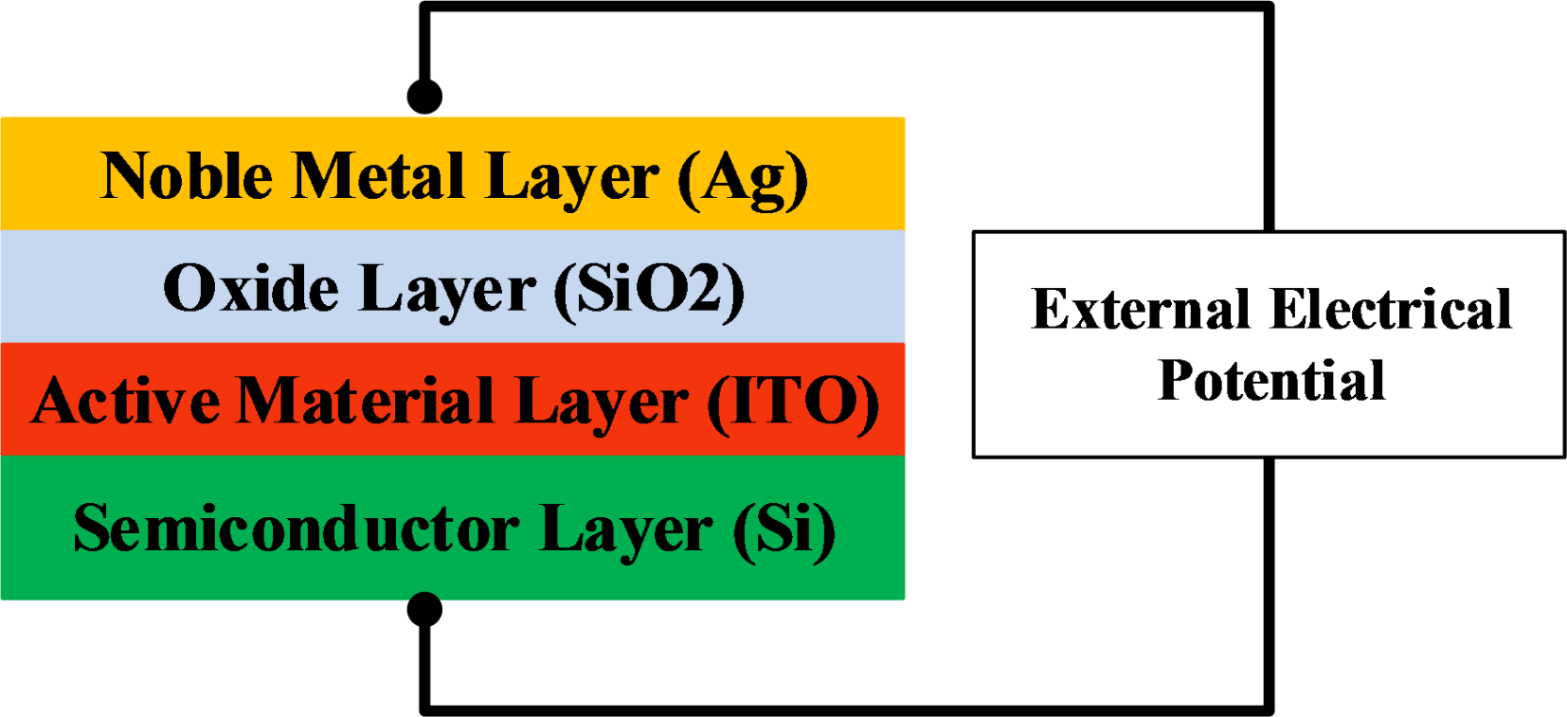
The 2D- YZ view of switching unit that controlling the filter mechanism.

The switching mechanism between the input, bus and output waveguides depends mainly the three switching units appeared in the structure. Each switching unit consists of four layers from slab waveguides; they arranged from bottom as shown in Fig. 2: a semiconductor (Si) in layer one, an active material which has electro-optic characteristics (ITO) in layer two, an oxide material (SiO2) in layer three, finally a noble metal (Ag) in layer four. The switching mechanism is achieved by changing the refractive index of the electro- optic active material (ITO) with an external electrical potential which leads to varying the effective index of the propagated optical mode and hence altering the modal overlap between the neighboring waveguides^22,23^.

The switching unit can function as a capacitor, as illustrated in Fig. 2. When a forward bias is applied to this capacitor—meaning a high voltage is applied to the Ag-layer and a low voltage to the Si-layer—a charging process is initiated. During this process, the unit absorbs electromagnetic waves from the waveguide, effectively blocking their transmission to adjacent waveguides. Conversely, in the absence of an applied voltage, the capacitor discharges, allowing electromagnetic waves to pass through to the neighboring waveguides.

The design parameters and optical properties of the ITO layer used in this work are based on previous studies^24–26^. Specifically, the refractive index of the ITO layer is 1.96 + i0.002 when the bias is set to zero volts, a condition referred to as the OFF state. When the bias is increased to 4 volts, the refractive index changes to 0.471 + i0.643, indicating the ON state. These optical properties are observed when the ITO layer has a thickness of 20 nm. The performance of the switching unit was initially evaluated by measuring the transmission in both the ON and OFF states, using the data showed in Table 1.

**Table 1:** The suggested design parameters of the proposed structure before optimization process.

Dimensions of Switching Units	Length: (L1 = L2 = L3 = 8 μm)
	Width of each switching unit (W1: 275 nm)
Each Si- arm	Width: (W2: 275 nm) and Height (340 nm)
Layers of each switching unit	Si layer with height equal to height of two Si- arms
	ITO layer with Height of 20 nm
	SiO_2_ layer with Height of 50 nm
	Silver layer with Height of 100 nm
Separated Gaps	The separated gaps between the switching units and Si waveguides are sited to be 150 nm, also the separated gaps between the switching units themselves are sited to 50 nm

The structure proposed in Fig. 1 is considered a multi- functional device that realize the function of four filter’ types (LPF, HPF, BPF, and BRF) which are pivotal in various optical communication applications. The structure is excited by a transverse magnetic (TM) polarized light at the input port in X- direction (propagation direction) and deliver the output at the output port. According to the Lumerical 3D-FDTD Solutions tools and after placing power monitors at the input/output ports, the device performance can be tested via measuring some parameters such as: wavelength spectrum (transmittance at output port), power consumption, optical losses (transmittance at isolated ports), footprint, and full width half maximum (FWHM). The transmittance of output and isolated ports can be determined through Eqs. (1 and 2):

1 $$Transmittan ce_{{Output\;Port}} = \left( {\frac{{Power_{{Output\;Port}} ~~}}{{Power_{{Input\;Port}} ~}}} \right)$$

2 $$Transmittan ce_{{Isolated\;Ports\left( {A,\;B,\;C,\;D} \right)}} = \left( {\frac{{Power_{{Isolated\;Ports\left( {A,\;B,\;C,\;D} \right)}} ~~}}{{Power_{{Input\;Port}} ~}}} \right)$$

3 $$Full\;Width\;Half\;Maimum\;\left( {FWHM} \right) = \left( {\lambda _{1} - ~\lambda _{2} } \right)$$

Where; ${\varvec{\lambda}}_{1}$ and ${\varvec{\lambda}}_{2}$ are the cut-off wavelengths at which the transmittance equals half of its maximum values.

### The performance of four’ type plasmonic filters

The proposed structure can model the four’ types depending on the switching case of three switching units appeared in the structure. Table 2 illustrates the switching case of three switching units that applied during the simulation process to realize the interested filter’ type. For example; to apply the BPF’ function, the three switching units are considered in the ON state at the same time. The same applies to the rest of the cases as shown in the table.

**Table 2:** The filter’ type according to the case of switching units.

Filter’ type	Switching Unit 1	Switching Unit 2	Switching Unit 3
BPF	ON	ON	ON
HPF	ON	OFF	ON
LPF	OFF	OFF	OFF
BRF	OFF	ON	OFF

The performance of a plasmonic filter is influenced by several design parameters, including the dimensions of the three Si waveguides, the gaps between these waveguides, the dimensions of the switching units, the gaps between the switching units, the dimensions of the slab waveguides used in each switching unit, and the materials utilized. To enhance the filter’s performance, an optimization algorithm can be employed, particularly a multi-objective optimization approach that addresses conflicting design goals, such as maximizing transmission efficiency, minimizing bandwidth, and reducing the overall footprint.

The initial simulations begin with design parameters sourced from previous research, along with parameters specifically proposed for this work. Following this, optimization is applied to further improve the filter’s performance. The final stage of the optimization process yields the optimized design parameters, which are detailed in Table 3.

**Table 3:** The optimized design parameters used in the final stage of the simulation.

Dimensions of Switching Units	Length: L1 = L3 = 3 μm Length: (L2 = 6 μm)
	Width of each switching unit (W1: 300 nm)
Layers of each switching unit	Si layer with Height of 300 nm
	ITO layer with Height of 20 nm
	SiO_2_ layer with Height of 20 nm
	Silver layer with Height of 100 nm
Si’ Waveguides	The three Si WGs printed on the substrate SiO2 are considered to be 300 nm in width and 300 nm in depth
Separated Gaps	All separated gaps between the switching units and Si waveguides, also the gaps between the switching units themselves are sited to 50 nm

**Fig. 3:**
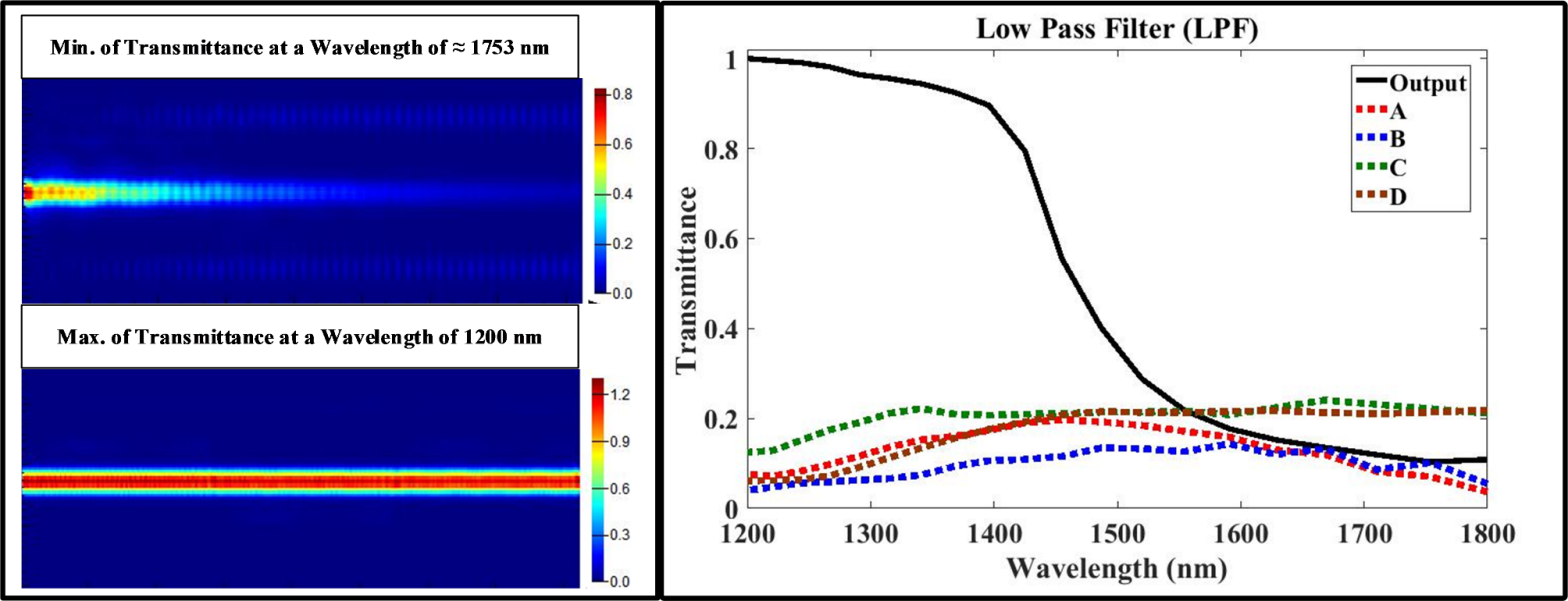
On the right-hand side, the transmission spectrum of low pass filter (LPF); on the left hand-side, the electric field intensity distribution of plasmonic LPF over XY plane at the wavelength of ≈ 1753 nm which satisfies minimum transmission in the upper part and the wavelength of 1200 nm which satisfies maximum transmission in the lower part).

**Fig. 4:**
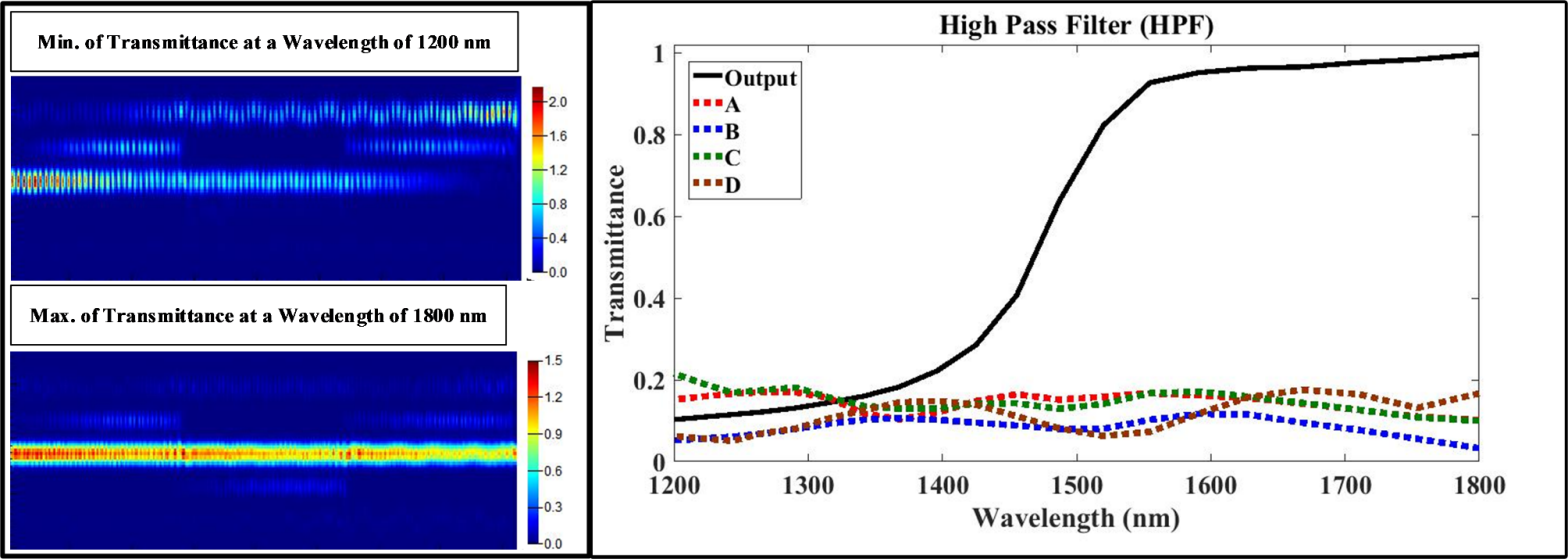
On the right-hand side, the transmission spectrum of high pass filter (HPF); on the left hand-side, the electric field intensity distribution of plasmonic HPF over XY plane at the wavelength of 1200 nm which satisfies minimum transmission in the upper part and the wavelength of 1800 nm which satisfies maximum transmission in the lower part).

**Fig. 5:**
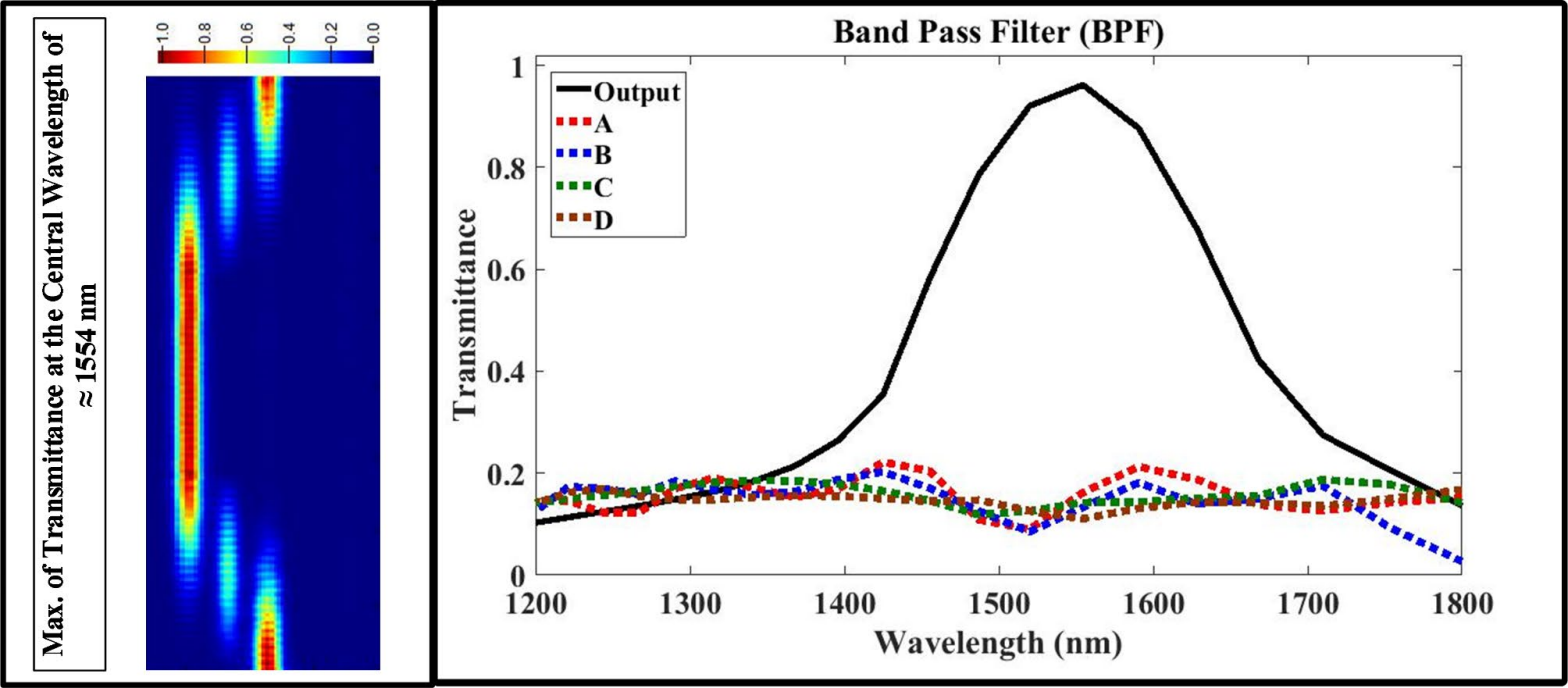
On the right-hand side, the transmission spectrum of band bass filter (BPF); on the left hand-side, the electric field intensity distribution of plasmonic BPF over XY plane at the central wavelength of 1554 nm which satisfies maximum transmission.

**Fig. 6:**
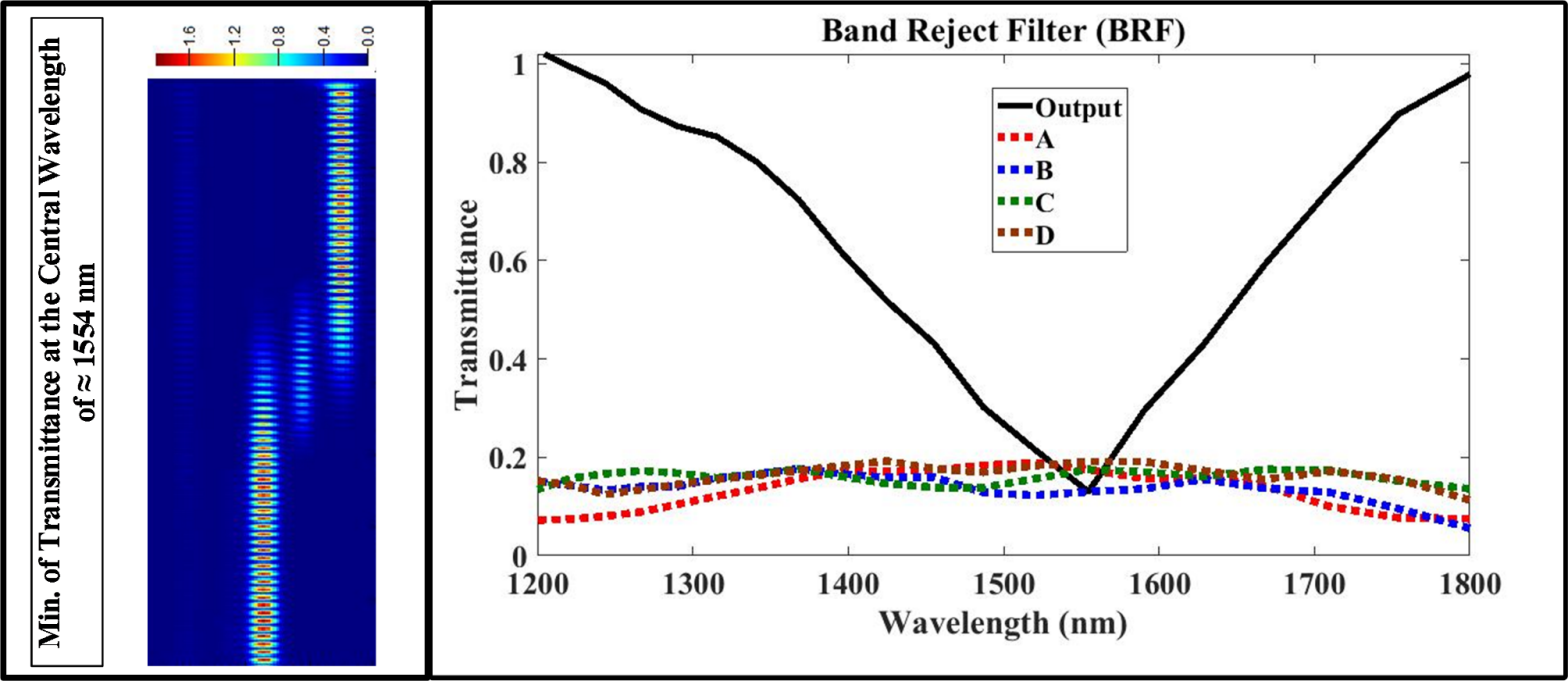
On the right-hand side, the transmission spectrum of band reject filter (BRF); on the left hand-side, the electric field intensity distribution of plasmonic BRF over XY plane at the central wavelength of 1554 nm which satisfies minimum transmission.

First, using the optimized design parameters shown in Tables 4 to model the multi-functional structure using Lumerical FDTD Solutions’ software. Second, exciting the structure with the fundamental transverse magnetic optical mode with extended wave length range from 1200 nm to 1800 nm. Finally, putting the measured monitors at input/output ports to test the performance of proposed structure using Eqs. (1, 2, 3, and 4) with aid of data announced in Table 2. Figures 3, 4, 5 and 6 show the analyzed results of transmittance at the output and isolated ports over the selected wavelength range, which verify the filtering’ function of four filters (LPF, HPF, BPF, and BRF).

According to Figs. 3 and 4, the transmittance of the proposed structure verifies the filtering function of LPF and HPF with cut-off frequency of 1554 nm, and this is located in the telecommunication window of the electromagnetic spectrum. According to the simulations, the optimization process minimizes the band width of passing wavelength range while maximize its transmittance at the output port and blocking the transmittance at the isolated ports (A, B, C, and D). According to Figs. (5 and 6), the transmittance of the proposed structure verifies the filtering function of BPF and BRF with central frequency of 1554 nm, and this is also located in the telecommunication window of the electromagnetic spectrum.

The transmittance over the selected range satisfies good results at pass bands over 98% at output port and less than 2% at isolated ports (A, B, C and D). During the optimization process, we target reducing the FWHM in the resonant modes (sharping the resonance curves) for both BPF and BRF which considered another way to improve the filter performance. After using the Eq. (3), the estimated value of FWHM for the BPF is ≈ 216 nm, but equals to ≈ 223 nm for the BRF; and this is considered a useful feature for this multi-functional structure. Now we calculate and display the electric field intensity distribution, |E|, at the cut-off and central frequencies for the four filter’ types. Figures 3, 4 and 5, and 6 shows also the distribution of the electric field at the cut-off and central wavelengths of four filter’ types.

## Conclusion

In conclusion, the work proposed modeling a high performance multi- functional active plasmonic structure that realizes the four’ type filters (LPF, HPF, BPF, and BRF) with aid of an electro-optic material (ITO) which controlling the transmission of optical mode from input to output ports after sandwiching it during the layers of three switching units appeared in the direction of optical propagated mode. From the numerical results of the *Lumerical 3D-FDTD Solutions tools* and analyzing the obtained data results using the equations mentioned in Sect. (2.1.); the proposed structure satisfies good results over the selected range, where the transmittance at pass bands over 98% at output port and less than 2% at isolated ports (A, B, C and D). The cut-off and central wavelengths of four’ type filters are located in the telecommunication window of the electromagnetic spectrum. The structure shows another useful feature that improve the filter performance; this is the estimated value of FWHM for the BPF which equals to ≈ 216 nm, and ≈ 223 nm for the BRF and this is considered a moderate value for a multi-functional structure. The proposed structure has an ultracompact size or a small foot-print of $\approx21$ μm^2^. Therefore, the structure may find significant applications in highly integrated dense wavelength division multiplexing systems. The feature of modeling four’ type filters using one structure will significantly reduce costs and economic efficiency for the production.

## Data Availability

The datasets analyzed during the current study available from the corresponding author on reasonable request.

## Declarations

### Software

The version to the used software: Lumerical FDTD Solutions 8.12.501 for x64. The link to the used software: https://www.lumerical.com.
